# Ruptured bilateral brain arteriovenous malformations in a young woman with early pregnancy: a case report

**DOI:** 10.1186/s13256-023-03946-6

**Published:** 2023-05-27

**Authors:** Ng’weina F. Magitta, Emmanuel M. Sindato, John R. Meda, Hasna N. Toroha, Alfred J. Meremo

**Affiliations:** 1 Department of Internal Medicine, School of Medicine & Dentistry, College of Health and Allied Sciences, University of Dodoma, Dodoma, Tanzania; 2 Department of Internal Medicine, Benjamin Mkapa Hospital, Dodoma, Tanzania; 3 Department of Radiology, Benjamin Mkapa Hospital, Dodoma, Tanzania; 4 Department of Biochemistry & Department of Clinical Pharmacology, Mbeya College of Health and Allied Sciences, University of Dar Es Salaam, Mbeya, Tanzania

**Keywords:** Brain arteriovenous malformation, Intracranial hemorrhage, Seizures, Pregnancy, Spetzler-Martin classification

## Abstract

**Background:**

Brain arteriovenous malformations (AVMs) are rare congenital developmental vascular lesions, and often presents with symptoms upon rupture. The controversy exists as to whether pregnancy confers an increased risk of intracranial hemorrhage. The diagnosis of brain AVMs, in the absence of brain imaging, is challenging in resource-limited settings, particularly in sub-Saharan Africa.

**Case presentation:**

A 22-year old black African woman, primigravida at 14 weeks of gestation, presented with a history of persistent throbbing headache which was treated at primary health care facilities with analgesics and anti-migraine medications without relief. She later developed severe headache 2 weeks prior to admission and one-day history of serial partial generalized tonic–clonic seizures which were followed by post-ictal confusion and persistent right upper limb weakness. Initial evaluation revealed her to be pregnant and she later underwent a brain magnetic resonance angiography (MRA) at a university teaching hospital which revealed bleeding bilateral parietal AMVs with intracerebral haematoma and associated perilesional vasogenic oedema. The patient was managed conservatively using antifibrinolytic drugs and prophylactic anti-seizure drugs. Seven months later, she underwent a control brain MRA which revealed resolution of intracranial haematoma and associated vasogenic oedema and had her seizures well controlled. The headache had subsided and the pregnancy was allowed to continue to term under close obstetric and neurological observation. On follow up visits she reported episodes of nasal bleeding which upon ENT examination revealed nasal AVMs, suggesting the diagnosis of hereditary hemorrhagic telangiectasia (HHT).

**Conclusion:**

AVMs are rare but should prompt suspicion in young patients with atypical Central Nervous System (CNS) manifestations without evident underlying causes.

## Introduction and literature review

Brain arteriovenous malformations (AVMs) are rare congenital developmental vascular lesions. The prevalence of AVMs is estimated at 0.1–0.4% globally [1], with few reported cases from Africa. AVMs underlie an estimated 1–2% of all strokes, 3% of strokes in young adults, and 9% of subarachnoid hemorrhage (SAH) [2]. The majority of hospitals in sub-Saharan Africa, except for a few specialized centres, do not have access to advanced brain imaging, which hampers both the diagnosis and staging of patients as well as planning for management approaches for brain pathologies, including AVMs.

AVMs often do not cause major problems until they rupture, except when impacts pressure on adjacent sensitive sites. The ruptured brain AVMs lead to intracranial hemorrhage (ICH) with consequent mass effect, vasogenic oedema and variable symptomatology [3]. The symptoms include headache, seizures, and focal neurological deficits, while in a significant number of cases; AVMs may be identified as *incidentilomas* during diagnostic workups for other neurological conditions. In the setting of shortage of diagnostic capacity, patients with AVMs are often misdiagnosed or misclassified and managed for stroke of unknown origin, epilepsy or even migraine headaches. Interestingly, patients with non-ruptured AVMs may present with subtle neurological symptoms corresponding to the location and size of the lesion. A number of risk factors have been reported to increase the risk of rupture of AVMs, with pregnancy remaining largely equivocal [4].

The aetiology of AVMs remains elusive. However, the majority of the lesions are sporadic, harboring mutations of RAS, BRAF, MEK1/2 and other downstream molecules involved in the mitogenic pathways including mitogen activated protein kinase (MAPK). AVMs are therefore, classified as ‘RAS*opathies*’[5]. A recent in vitro study by Lara Al-Olabi revealed that mutations in MAPK pathway—encoding genes lead to aberrant signaling and disruption of vascular endothelial tube formation and increased tendency to vasculopathy [6]. Moreover, murine knock out experiments of Leucine Zipper Transcription Regulator-1 (LZTR1) gene—which code for a *kelch* domain-containing protein—results in over-expression of RAS with subsequent aberrant activation of MAPK signaling. In normal cells, LZTR1 acts as a negative regulator of MAPK pathway through polyubiquitination of specific lysine residues (K48, K33 and K66), tagging the RAS-GTPases for proteosomal degradation [7]. Intriguingly, Tao Hong, et al. have demonstrated a high prevalence of KRAS/BRAF somatic mutations in the brain and spinal cord AVMs, with p.G12D KRAS mutation alone accounting for 52.4% of all mutations in brain AVMs [8], thus, highlighting the potential for a targeted monoclonal antibody therapy. Inadvertently, in Africa, this tailored therapy is often limited by lack of expertise and resources necessary for mutational analysis.

Noteworthy, a small proportion of patients have familial form of AVMs which typically occur within the myriads of hereditary hemorrhagic telangiectasia (HHT), *alias* Osler-Weber-Rendu syndrome – an autosomal dominant, multisystem disease, whose diagnosis is based on Curaçao criteria, as described elsewhere [9]. In HHT, mutations of cognate genes results in aberrant transforming growth factor- beta (TGF-β) signaling pathway and subsequent over-expression of vascular endothelial growth factor (VEGF) which leads to the formation of abnormal and fragile vascular beds [10].

The management of brain AVMs is guided by severity grade scales, for instance, by Spetzler-Martin (S-M) classification—which is dependent on the size of the *nidus*, location in the eloquent or non-eloquent brain site, and presence of deep venous drainage—with a higher scale corresponding to a higher risk of surgical morbidity and neurological deficit. In patients with non-ruptured brain AVMs, surgical intervention has not been shown to be superior to conservative management in terms of outcomes [11–13].

This patient presented with recurrent headache probably prior to the rupture of brain AVMs. Thus, the case is unique in the settings of limited resources where prompt brain imaging services are not readily available. Moreover, the case represents a relatively rare condition and is an eye opener to the clinicians to have a high index of suspicion when a young patient presents with recurrent headache not responsive to the usual analgesics. Early suspicion and diagnosis of brain AVMs could help to mitigate untoward consequences associated with their unpredicted rupture.

## Case presentation

### Demographic details and medical history

A 22-year old black African woman, single, primigravida at 14 weeks gestation, otherwise apparently well, presented with a 1-month history of unilateral left-sided throbbing headache, without facial radiation, which was not relieved by analgesics—including paracetamol 1 g q8h and ibuprofen 400 mg q8h per oral—and anti-migraine drugs-vasograin—ergotamine tartarate 1 mg, caffeine 300 mg, paracetamol 250 mg, prochlorperazine maleate 2.5 mg q6h per oral, at a primary care facility. On enquiry, the patient reported a history of nausea associated with early morning non-projectile vomiting. She later developed severe headache 2 weeks prior to admission and one-day history of serial seizures which were characterized by jerky movement of the right arm which progressed to generalized tonic–clonic seizures accompanied by eye rolling without loss of consciousness, each lasting 2 to 3 min, followed by confusion and persistent right arm weakness. These convulsions were not preceded by abnormal physical sensation or emotional disturbances. There was no history of urine or fecal incontinence.

She did not report any history of fever, head trauma, ear or nasal discharge, easy bruising and bone pains. Moreover, she did not report any visual or speech disturbances, hearing loss or dizziness. She had no family history of similar illness, epilepsy or seizure disorders. She admitted to have a regular consumption of pork at casual food vendor outlets. She denied any history of cigarette smoking and alcohol consumption. Her past medical history was unremarkable, without a history suggestive of syphilis infection and any perceived increased risk of HIV infection. She was a primigravida at 14 weeks of gestation, without any untoward pregnancy complications and she denied any history of abortions. The review of other systems was non-contributory. She was the third born in a family of four siblings, without any family history of any known genetic condition or allergies. She was working as a cashier at a small company after dropping out of  the university due to lack of tuition fee. Intriguingly, during the follow up visit the patient reported to have two episodes of epistaxis. The ear, nose and throat (ENT) examination revealed actively bleeding small tuft of blood vessels in the right nasal mucosa.

### Clinical findings

The patient was evaluated, at the emergency department, and found to be at 14-week pregnancy. She was fully alert, with warm extremities, not dyspneic, not pale, not icteric, and afebrile, without superficial lymphadenopathy. She did not have skin rashes, freckles, or  lower limb oedema. ENT examination was unremarkable on admission.

The patient had stable vitals, with a blood pressureof 110/60 mmHg, pulse rate  of 101 beats per minute, temperature of 36.1°C and oxygen saturation of 99 percent on room air.

The CNS examination; She was fully alert, oriented to time, place and person, had intact short and long-term memory with normal speech and language. All cranial nerves were intact and the patient did not have anisocoria and had normal sized pupils with normal reaction to light. Examination of upper and lower limbs revealed normal muscle bulkiness and tone in all muscle groups except for  reduced power of grade 3 out of 5 on the right upper limb. Both superficial and deep tendon reflexes were normal. The Babinski sign was negative and the clonus was not sustaining. The examination of all sensory modalities was intact in all dermatomes of the upper and lower limbs. The coordination, balance and gait were all normal. Examination of the spine was essentially normal. Other systemic examinations were unremarkable.

### Timeline

The index patient was evaluated in May, 2022 during her initial visit where she presented with a history of headache, convulsions and right upper limb weakness. She underwent brain MRA imaging and other evaluation which revealed ruptured bilateral parietal AVMs with intracerebral hemorrhage and associated peri-lesional oedema. At 4 weeks, she was re-evaluated and at 7 months, December. 2022, a repeat MRA was performed. The patient was managed conservatively with a good clinical and radiological response.

### Diagnostic assessment

#### Magnetic resonance angiography (MRA)

**Fig. 1:**
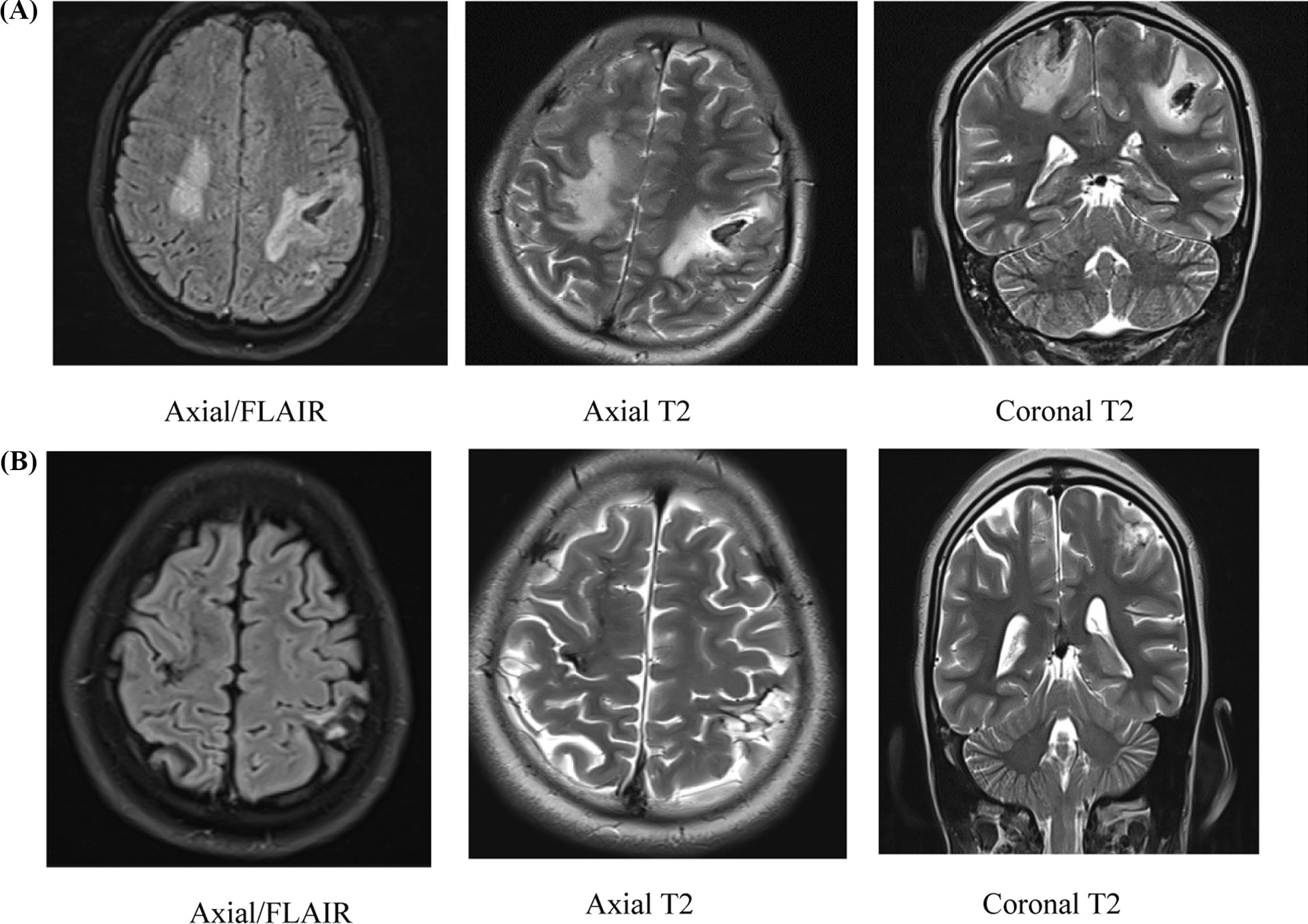
**A** Dark fluid and T2W sequences, axial and coronal images showing dilated tortuous tubular and serpentine structures of signal void appearance in T2/FLAIR sequences in the bilateral fronto-parietal cerebral lobes with surrounding notable vasogenic edema. **B** Dark fluid and T2W sequences, axial and coronal images: Reduced tortuous tubular and serpentine structures of signal void appearance in T2/FLAIR sequences are noted in the bilateral parietal cerebral lobes. In Axial T2 and Coronal T2, complete resolution of right parietal vasogenic edema and reduced surrounding vasogenic edema seen

**Fig. 2:**
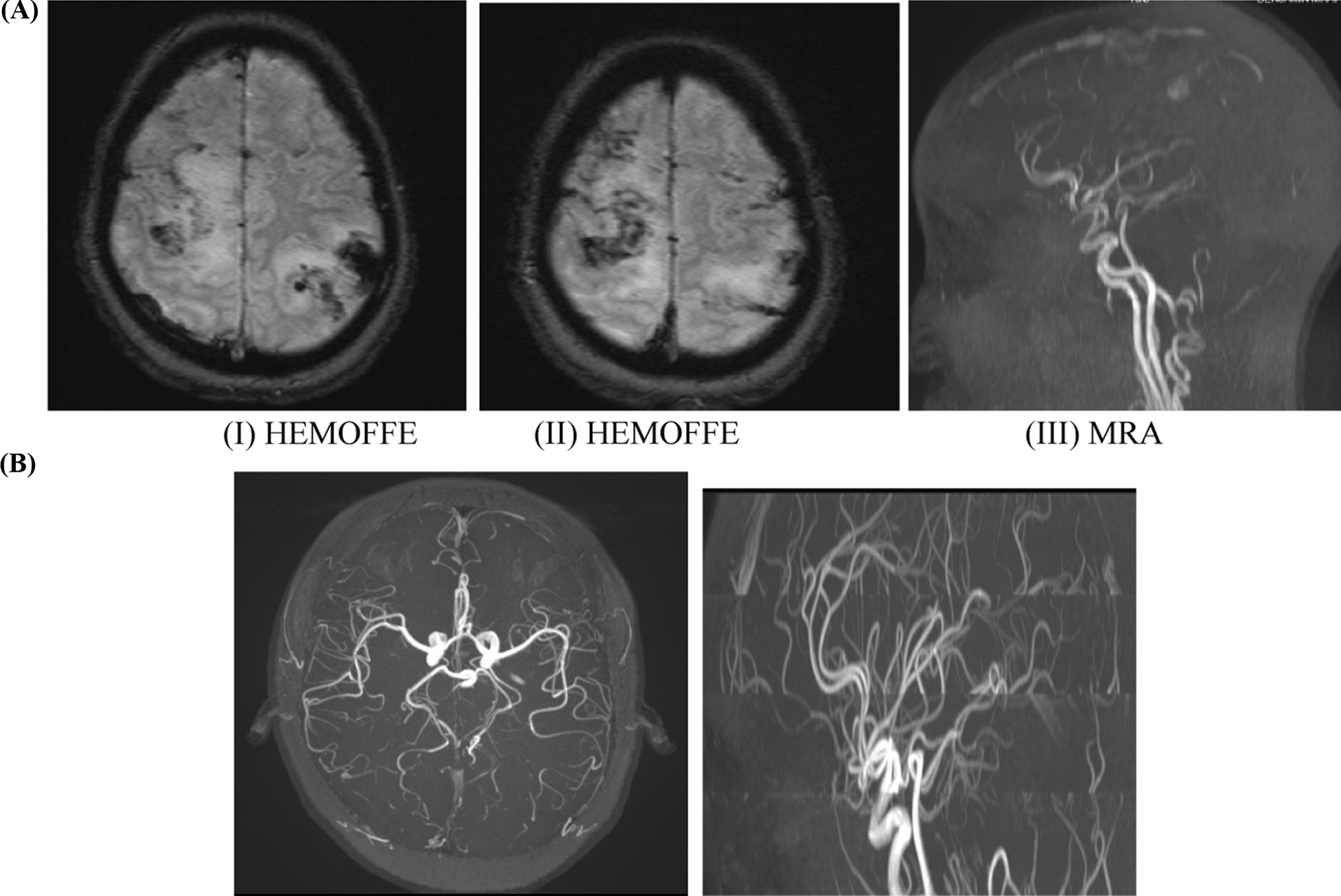
**A** Dark fluid and T2W sequences, axial and coronal images showing bilateral *nidus* (‘‘*bag of worms’’*) showing susceptibility artifact on HemoFFE in the bilateral fronto-parietal regions (I, II). Right *nidus* demonstrated M3 cortical segment of middle cerebral artery (MCA) feeding artery and venous drainage to the superior sagittal sinus through cortical vein. Left *nidus* demonstrated M4 cortical segment of MCA feeding artery and venous drainage to superior sagittal sinus through the vein of Trolard (III). **B** MRA sequence, axial and sagittal images show: No obvious *nidus* of malformed vessels and no hemorrhage intracerebral noted

**Fig. 3:**
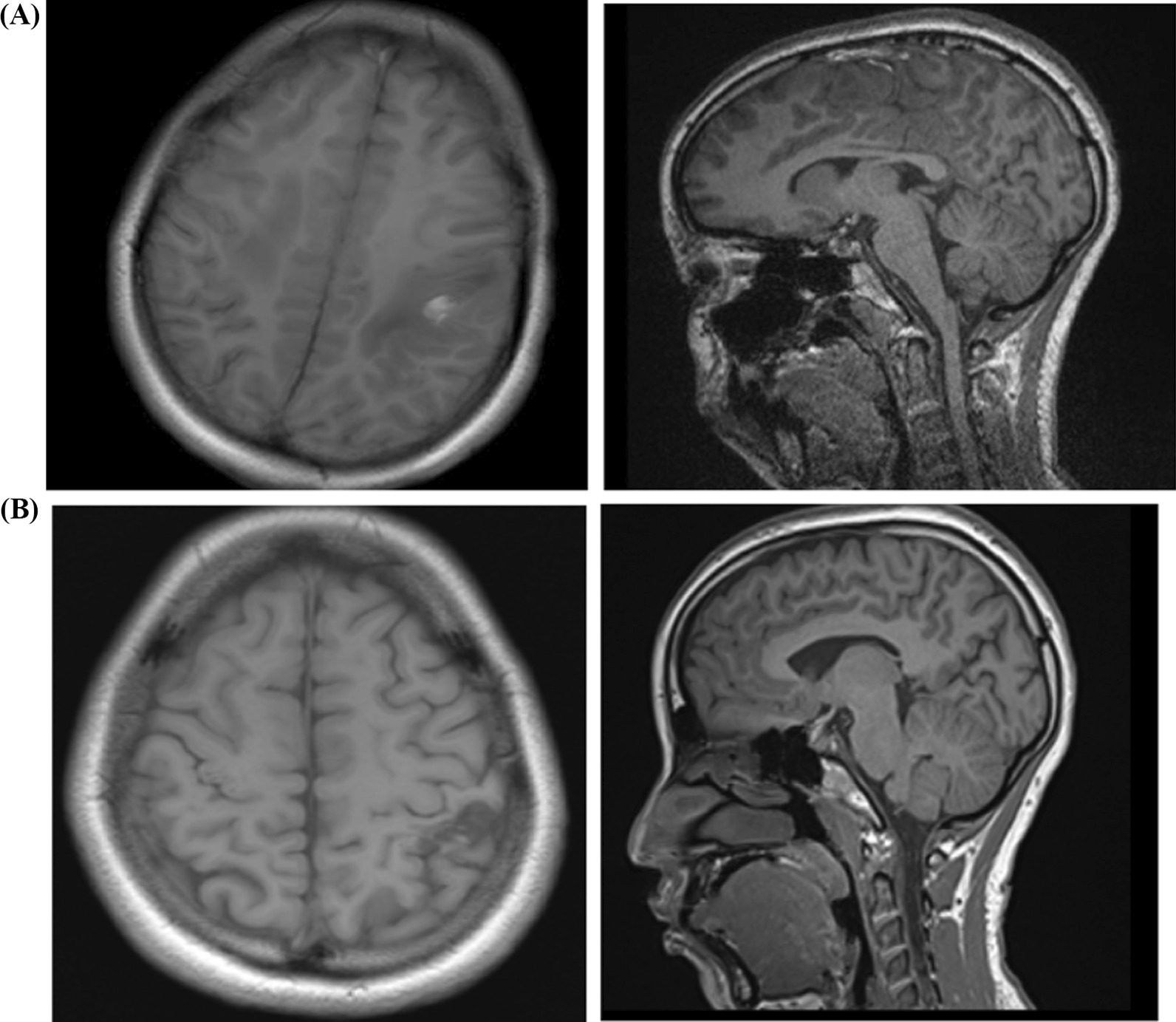
**A** Axial and sagittal T1W sequences, images showing focal hyperintense lesion with surrounding vasogenic edema noted in the left fronto-parietal region. Moderately dilated cortical veins are noted draining into the superior sagittal sinus. **B** Axial and sagittal T1W sequences images, respectively: Focal hypointense lesion with no surrounding vasogenic edema is noted in the left parietal region. No intracerebral hemorrhage noted

Brain MRA was performed which revealed a ruptured bilateral brain AMVs as depicted in Figs. 1A, 2A and 3A below. In brief, axial T2_ff2d_hemoFFE revealed multiple foci of low signal on the post central sulcus of both parietal lobes in keeping with AVMs. Associated hyper intense signal is also shown on axial sagittal and coronal T2WI. Axial T1WI shows intra-lesional foci of hyperintensity. The MRA/TOF images reveal connecting vessels particularly on the left side. These features are consistent with bilateral parietal lobe bleeding AVMs with associated brain oedema.

During follow up visits MRA imaging, (Figs. 1B, 2B and 3B) revealed intra-axial sub-cortical small tuft of vessels shown as aggregated linear signal voids involving the bilateral high convexity parietal region. Central feeders and venous drainage couldn't be adequately identified. No hemorrhage could be detected. These features were consistent with bilateral high convexity parietal AVM with resolved intracerebral hemorrhage.

Images at initial presentation (Figs. 1A, 2A and 3A).

Follow up visit images at 7 months (Figs. 1B, 2B and 3B).

### Therapeutic intervention

The patient was managed conservatively to control bleeding and seizures. She was managed conservatively with intravenous paracetamol 1 g q8h for three days, intramuscular injection trenaxamic acid 500 mg q8h per oral for three days, with repeat dose on the fourth week after presenting with epistaxis and anti-seizure drugs—lamotrigine 25 mg daily per oral for initial 2 weeks with dose escalation to the maintenance dose of 100 mg q12h daily per oral from the eighth week onwards and followed up with a repeat brain MRA on the seventh month. The pregnancy was allowed to continue to term under close obstetric and neurological monitoring.

## Follow up and outcomes

Brain MRA imaging performed at seven months revealed a good resolution of intracerebral haematoma and vasogenic oedema (Figs. 1B, 2B and 3B). On follow up visits she reported episodes of nasal bleeding which upon ENT examination revealed nasal AVMs which were managed conservatively, suggesting the diagnosis of hereditary hemorrhagic telangiectasia (HHT). The patient had normal cognitive function, with good orientation, as well as intact short and long-term memory, speech and language. The neurological examination was normal except for the reduced power of grade 4 out of 5 of the right upper limb. The patient was on prophylactic anti-seizure; lamotrigine 100 mg q12h daily. However, the patient reported recurrent episodic weakness of the right upper limb which resolves spontaneously. She is currently attending neurosurgery clinic for monitoring. She had uneventful delivery at full-term, to 3.4 Kilograms, healthy baby.

## Discussion

This patient presented with recurrent headache during the first trimester of pregnancy. However, in typical case scenarios, pregnancy-precipitated ruptured brain AVMs occurs during the second-to-third trimester. Interestingly, focal motor deficit which recur and resolve spontaneously at the same site is not typically observed in previously reported cases of ruptured brain AVMs. Thus, this case is unique and prompts a high index of suspicion for brain AVMs when a relatively young patient presents with atypical symptoms pertinent to a neurological disorder.

Brain AVMs are increasingly being reported in Africa owing to the improved diagnostic capacities in a few specialized centers. The index patient presented with a history of long-standing headache which was initially managed as migraine without resolution and subsequent presentation with nasal bleeding, which revealed nasal AVMs. The co-existence of peripheral (nasal) and brain AVMs in this patient is highly suggestive of HHT.

In the absence of rupture, brain AVMs rarely present with symptoms except when the lesion is large enough to cause pressure symptoms in eloquent sites. Increased haemodynamic changes associated with pregnancy is likely to contribute to increased vascular distension within the tortuous AVMs thus, resulting in remarkable pressure symptoms. Moreover, these pregnancy-induced haemodynamic changes might lead to subtle microvascular damage and bleeding which might lead to the exacerbation of pre-existing pressure symptoms [14]. The index patient presented with evidence for ruptured brain AVMs at 22 years of age, similar to previously reported studies. However, the risk of pregnancy to the rupture of brain AVMs remains controversial, with conflicting results. For instance, a study by Xi et al.indicated increased risk by 10.1% of AVMs bleeding during pregnancy [14], while other studies have previously indicated the contrary.

The size of the *nidus*, its location (eloquent *versus* non-eloquent site) and presence of deep venous drainage forms the basis for grading of ruptured AVMs and an indicator for the modality of management and prognostication, as applied in S-M scale. This patient had large, bilateral lesions, involving eloquent sites within both parietal lobes without deep venous drainage. The large size of AVMs could explain the long-standing headache that the patient initially presented with. Rupture of brain  AVMs with consequent development of cerebral oedema and associated mass effect could partly explain the herald of 2-week history of severe headache which was followed by tonic–clonic seizures. Nevertheless, the involvement of bilateral parietal lobes was not accompanied with gross motor deficit, except for the recurrent focal motor deficit involving the right upper limb. The patient did not exhibit any sensory deficit or any other disorder of language or speech—which are core functions of the parietal lobes. One possible explanation for the observed less sensorimotor deficit and sparing of language and speech could be the fact that the AVMs were not associated with significant deep venous drainage in which the excessive haemorrhage was occluded from deep structures and intraventricular involvement.

The major goal of interventional treatment include the reduction of the risk of AVM-related hemorrhage, seizures, and other neurologic impairments [15, 16]. However, the efficacy of interventional methods in improving outcomes remains debatable. Patients with a prior history of AVMs rupture and presence of associated aneurysms are at higher risk of subsequent hemorrhage [17]. Conservative medical management may be reasonable for older patients without additional risk factors or patients with an unacceptable risk of surgery. Brain AVMs with angiographic features suggesting an increased risk of recurrent hemorrhage, such as an associated aneurysm requires urgent intervention [17]. It has been observed that brain AVMs may undergo spontaneous regression in a subset of unpredicted patients [18, 19].

The main options for brain AVMs treatment are conservative medical management *versus* microsurgical excision or stereotactic radiosurgery; endovascular embolization is used as an adjunct intervention to surgery and stereotactic radiosurgery [20]. The index patient had a score of 4 (bilateral AMVs size > 6 cm = 3, and in an eloquent parietal lobes site = 1) on S-M scale which qualifies for conservative management. Conservative management entails the optimization of haemostatic *milieu* using antifibrinolytic agents, together with the control of associated seizures. During a 7-month follow up, a repeat MRA was done which revealed a significant regression of haematoma, resolution of cerebral oedema and relief of headache. Patients with ruptured brain AVMs are at an increased risk of future bleeding and seizures, thus it’s always prudent to consider long-term monitoring of patients and administration of prophylactic anticonvulsant drugs for long-term secondary prevention of seizure.

## Conclusion

AVMs are rare vascular malformations, of ill-elucidated congenital developmental origin. However, the increasing advancement in brain imaging is likely to improve the diagnosis and help to shed light on the actual burden of brain AVMs in Africa. Moreover, due to lack of expertise and resources, surgical intervention is often not readily offered whenever indicated. It is therefore critical to suspect ruptured brain AVMs in young patients who presents with ill-defined CNS symptoms without discernible causes. The diagnosis of brain AVMs prior to rupture provides an opportunity for adequate intervention as well as improved patient survival and long-term outcome.

## Data Availability

The raw data pertaining to this case report is available on reasonable request.
